# Autophagy Protects against Oxaliplatin-Induced Cell Death via ER Stress and ROS in Caco-2 Cells

**DOI:** 10.1371/journal.pone.0051076

**Published:** 2012-11-30

**Authors:** Yan Shi, Bin Tang, Pei-Wu Yu, Bo Tang, Ying-Xue Hao, Xiao Lei, Hua-Xing Luo, Dong-Zhu Zeng

**Affiliations:** 1 Department of General Surgery and Center of Minimal Invasive Gastrointestinal Surgery, Southwest Hospital, Third Military Medical University, Chongqing, China; 2 Department of Clinical Microbiology and Immunology, College of Medical Laboratory Science, Third Military Medical University, Chongqing, China; Technische Universitaet Muenchen, Germany

## Abstract

Oxaliplatin is included in a number of effective combination regimens used as first and subsequent lines of therapy for metastatic colorectal cancer. Accumulating evidence indicates that autophagy plays a significant role in response to cancer therapy. However, the role of autophagy in oxaliplatin-induced cell death remains to be clarified. In this study, we showed that oxaliplatin induced cell death and autophagy in Caco-2 colorectal cancer cells. The suppression of autophagy using either pharmacologic inhibitors (3-methyladenine, bafilomycin A1) or RNA interference in essential autophagy genes (ATG5 or Beclin1) enhanced the cell death and reactive oxygen species (ROS) production induced by oxaliplatin in Caco-2 cells. Blocking oxaliplatin-induced ROS production by using ROS scavengers (NAC or Tiron) decreased autophagy. Furthermore, numerous dilated endoplasmic reticula (ER) were present in oxaliplatin-treated Caco-2 cells, and blocking ER stress by RNA interference against candidate of metastasis-1 (P8) and C/EBP-homologous protein (CHOP) decreased autophagy and ROS production. Taken together, these data indicate that oxaliplatin activates autophagy as a cytoprotective response via ER stress and ROS in human colorectal cancer cells.

## Introduction

Colorectal cancer (CRC) is one of the most common malignancies and is a leading cause of cancer death [1], [2], [3]. When the disease is detected early, prior to locoregional or metastatic spread, the 5-year survival rate is greater than 90% [4]. However, for patients with metastatic CRC (mCRC), the 5-year survival rate is less than 10% [1]. Oxaliplatin, a third-generation platinum complex, is a key component of a number of effective combination regimens (e.g., with 5-fluorouracil and leucovorin) that are used as first and subsequent lines of therapy for mCRC. However, the mechanism of oxaliplatin-induced cell death remains to be elucidated.

Although it is thought that oxaliplatin compounds are cytotoxic because they inhibit DNA synthesis in cancer cells, a growing body of evidence suggests that autophagy also plays a significant role in its cancer therapeutic effects [5], [6], [7]. Autophagy is a stress-induced cell survival program in which cells under metabolic, proteotoxic, or other stresses remove dysfunctional organelles and/or misfolded/polyubiquitinated proteins by shuttling them to the lysosome for degradation via specialised structures called autophagosomes [8]. For tumour cells, autophagy is a double-edged sword: it can either prevent or promote carcinogenesis, and it can modulate the response to anticancer therapy, including drug-induced cell death [9], [10]. Thus, it is necessary to clarify the role of autophagy in oxaliplatin-induced cell death.

Reactive oxygen species (ROS) have recently emerged as promising targets for anticancer drug discovery [11]. ROS are highly reactive oxygen free radicals or non-radical molecules that can be generated by multiple mechanisms; the nicotinamide adenine dinucleotide phosphate oxidases (NOX) and mitochondria are the major sources of ROS in the cell [12]. A recent investigation found that ER dysfunction could be either a source or a consequence of increased ROS generation [13]. These ROS are important multifaceted signalling molecules that can regulate a number of cellular pathways and thus play critical roles in determining cell fate [12]. Depending on the cell type and context, autophagy may play a critical role in regulating ROS-mediated survival or death in cancer cells [14].

**Figure 1:**
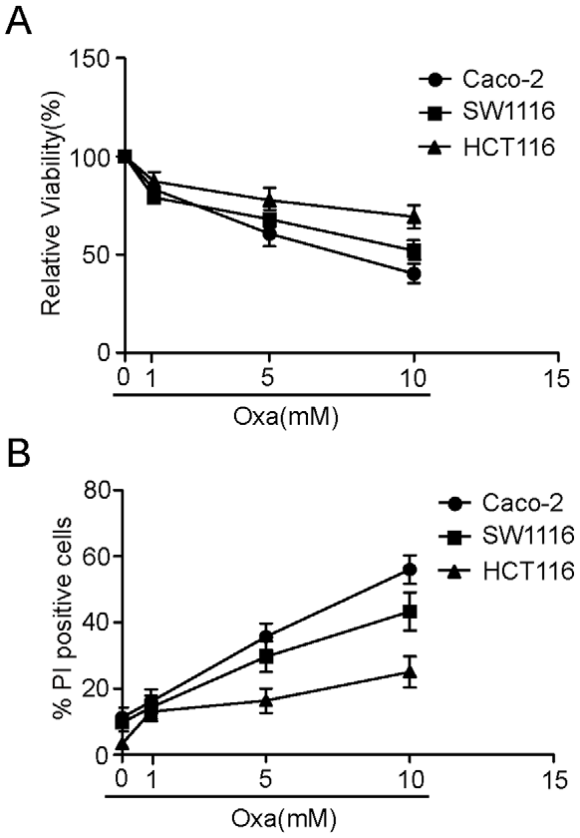
Oxaliplatin induces cell death in human colorectal cancer cells. ( A) The effect of oxaliplatin on the viability of human colorectal cancer cells. Caco-2, SW1116 and HCT116 cells were treated with the indicated concentrations of oxaliplatin for 24 hrs. Cell viability was assessed using MTT. (B) The three types of colorectal cancer cells were either untreated or treated with the indicated concentrations of oxaliplatin for 24 hrs, stained with PI and analysed by flow cytometry. The percentage of cells positive for PI is presented in the bar charts. The data shown are representative of three independent experiments.

**Figure 2:**
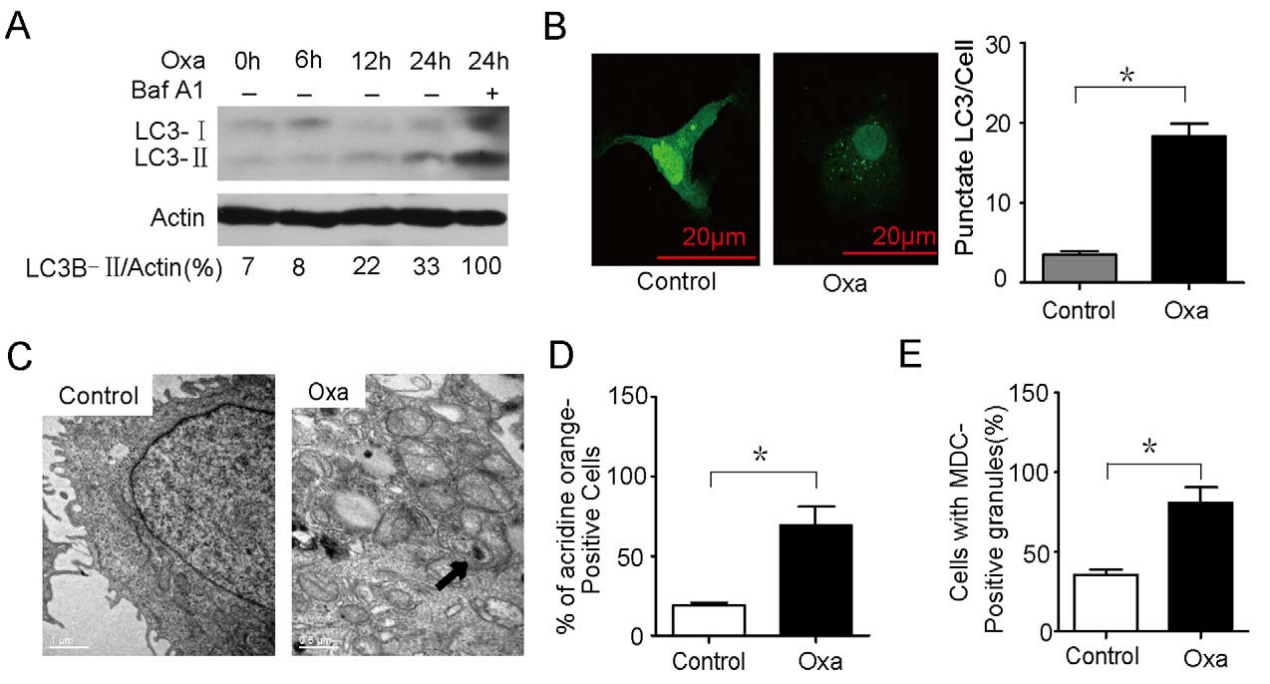
Autophagy is induced in Caco-2 cells after oxaliplatin treatment. ( A) Oxaliplatin induced incomplete autophagic flux in Caco-2 cells. Caco-2 cells were treated with oxaliplatin (10 µM) for 24 hrs in the presence of Baf A1 (10 nM). (B) Caco-2 cells were transfected with a plasmid expressing GFP-MAP1LC3B. After 24 hrs, the cells were exposed to oxaliplatin for 24 hrs. Cells were visualised by confocal microscopy immediately after fixation. The number of GFP-MAP1LC3B puncta in each cell was counted. (C) Ultrastructural changes in oxaliplatin-treated AGS cells. The control shows samples without oxaliplatin treatment. Closed arrows indicate autophagosomes. (D and E) Caco-2 cells were treated with 10 µM oxaliplatin for 24 hrs and stained with 1 mg/ml acridine orange or 50 mM MDC for 15 min. After incubation, cells were immediately analysed by flow cytometry. The bar chart demonstrates an increase in mean fluorescent intensity. The asterisks denote significant differences from controls (*P<0.05). Experiments were performed in triplicate, and all replicates showed similar results.

The role of autophagy in oxaliplatin-induced cell death in human colorectal cancer cells has not been explored. In this study, we investigated whether the autophagic machinery could be activated in Caco-2 cells after oxaliplatin treatment and determined that autophagy protects against oxaliplatin-induced cell death via reactive oxygen species production in Caco-2 cells. In addition, we assessed the role of ER stress as a trigger of ROS production during oxaliplatin treatment.

## Materials and Methods

### Antibodies and Reagents

The GFP-MAP1 LC3B plasmid was kindly provided by Dr. Tamotsu Yoshimori (Department of Cell Biology, National Institute for Basic Biology, Presto, Japan). Oxaliplatin (O9512), N-acetyl-L-cysteine (NAC, A9165), 4,5-dihydroxy-1,3-benzene disulfonic acid (Tiron, 172553), 3-methyladenine (3-MA, M9281), bafilomycin A1 (Baf A1, B1793), Thapsigargin (Thap, T9033) and rapamycin (Rapa, R8781) were purchased from Sigma; antibodies against MAP1 LC3B (L7543) and ATG5 (WH0009474 M1) were also obtained from Sigma. The antibody against Beclin1 (612112) was obtained from BD Transduction Laboratories, Inc. (Beverly, MA), and antibodies against Actin (sc-10731) and P8 (sc-23283) were obtained from Santa Cruz Biotechnology. The antibody against CHOP/GADD153 (#2895) was purchased from Cell Signaling Technology.

### Cell Culture

The human colorectal cancer cell lines (Caco-2, SW1116 and HCT116) were bought from cell bank (Chinese Academy of Sciences), and were separately cultured in DMEM media (Gibco, #11965-092), L-15 media (GIBCO, #41300039) and McCOY’s 5A media (SIGMA, #M4892) supplemented with 10% foetal bovine serum (Gibco, #10099-141) and 100 U/ml penicillin/streptomycin (Gibco, #15140-122) in a humidified incubator containing 5% CO2 at 37°C.

### MTT Assay

MTT assays were performed in a 96-well plate according to the manufacturer’s instructions (Sigma). After the indicated treatments, cells were incubated with MTT at a final concentration of 5 mg/L. After 1–2 hrs, the medium was removed, and the cells were dissolved in MTT solubilisation solution (Sigma). Absorbance at 590 nm (*A*590) was determined for each well using a microplate reader (Bio-Rad). After subtracting the background absorbance, the *A*590 of the treated cells was divided by that of the untreated cells to determine the percentage of viable cells.

**Figure 3:**
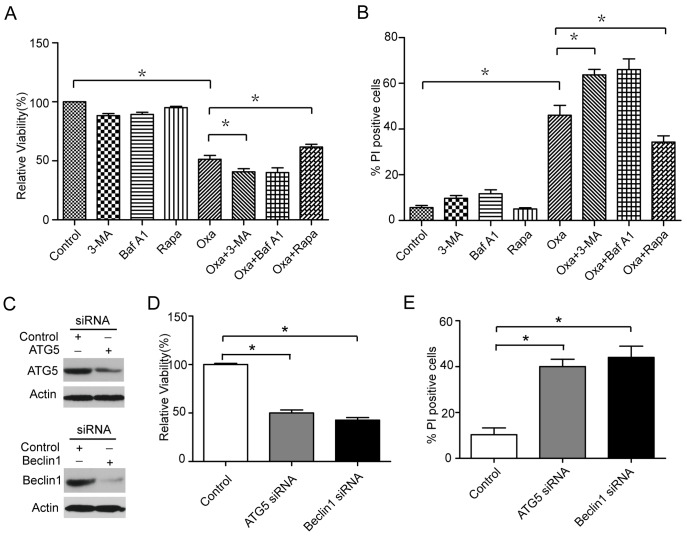
Autophagy protects against oxaliplatin-induced cell death. (A and B) Caco-2 cells were treated with 100 nM rapamycin, 5 mM 3-MA, 10 nM Baf A1, 10 µM oxaliplatin, or the indicated combinations for 24 or 48 hours. The percentage of dead cells was determined using the MTT assay or cell death assay. (C) Detection of the inhibition efficiency of siRNAs against ATG5 and Beclin1. Caco-2 cells were transfected with siRNAs targeting ATG5 and Beclin1 (100 nM each) for 24 hrs, and the protein levels of the two targets were evaluated by western blot. (D and E) MTT and cell death assays measuring the oxaliplatin-treated cell death ratio after transfection with siRNAs against ATG5 and Beclin1. AGS cells were transfected with Beclin1, ATG5 or a control siRNA at 100 nM for 24 hrs followed by treatment with oxaliplatin (10 µM). The data shown are the means and SD of at least three independent experiments. *, *P*<0.05.

**Figure 4:**
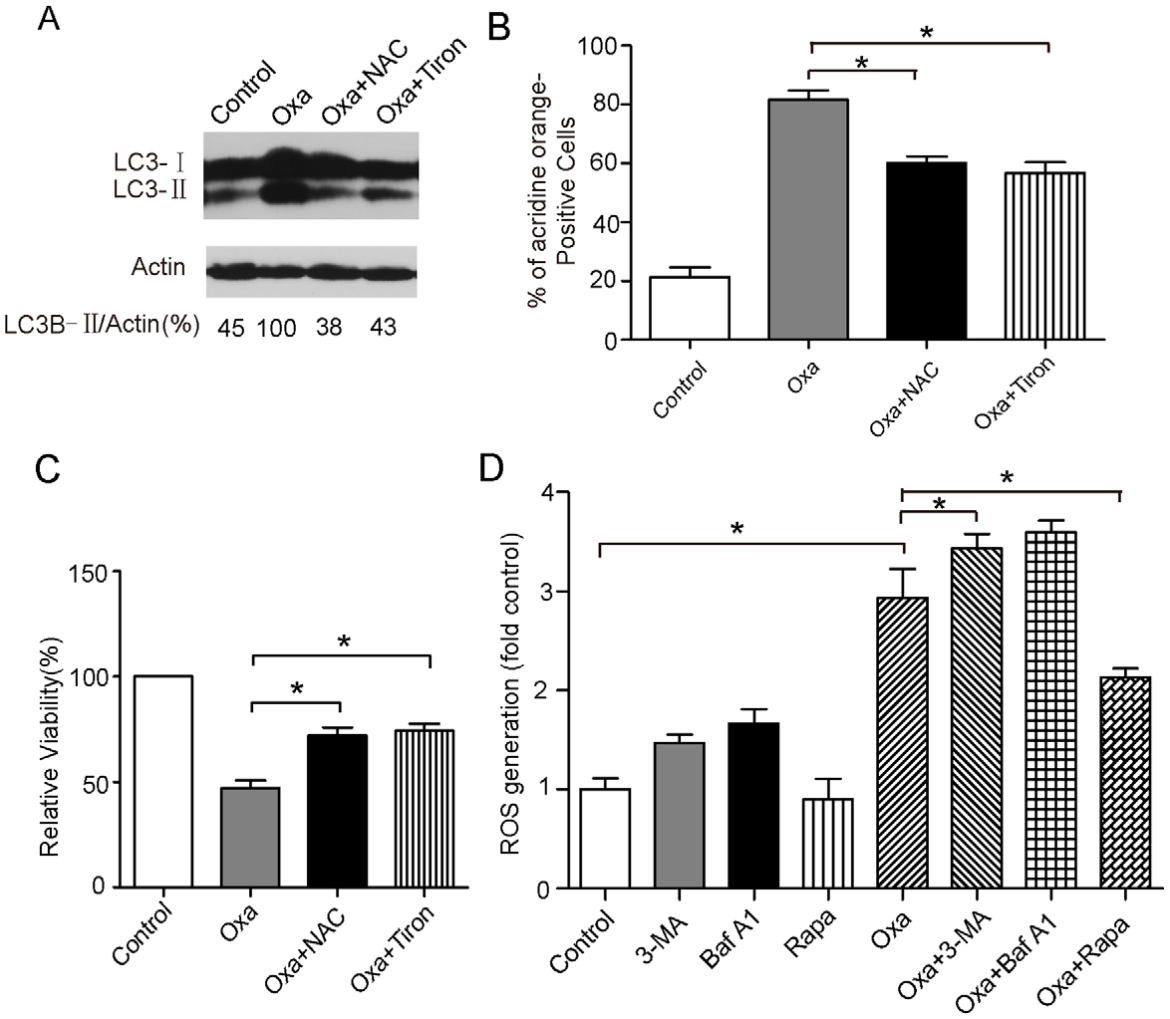
Oxaliplatin-induced autophagy is dependent on ROS. (A, B and C) Caco-2 cells were pretreated with 10 mM N-acetyl-cysteine (NAC) or 1 mM Tiron for 6 hours. Following pretreatment with NAC or Tiron, cells were exposed to 10 µM oxaliplatin for 24 hours. MAP1LC3B-II expression was measured by western blot assay. The cells that were positive for autophagosomes were detected using an acridine orange staining assay. Cell viability was assessed using MTT. (D) Caco-2 cells were treated with 100 nM rapamycin, 5 mM 3-MA, 10 nM Baf A1, 10 µM oxaliplatin, or the indicated combinations for 24 or 48 hours. Cellular ROS generation was determined using 2′, 7′-dichlorofluorescein diacetate in conjunction with flow cytometry. The data shown are the means and SD of at least 3 independent experiments. *, *P*<0.05.

### Acridine Orange Staining

In acridine orange-stained cells, the cytoplasm and nucleus appear bright green and dim red, respectively, and acidic compartments appear bright red [15]. The intensity of the red fluorescence is proportional to the degree of acidity. After receiving the specified treatments, cells were incubated with acridine orange solution (1 mg/ml) for 15 min in drug-free medium at 37°C and washed with PBS. Then, cells were trypsinised and analysed by flow cytometry using a FACScan cytometer and CellQuest software, as previously described. Statistical analyses were performed as described above.

### Monodansylcadaverine (MDC) Staining

Monodansylcadaverine (MDC) staining was used to quantify the induction of autophagy in Caco-2 cells treated with oxaliplatin. Following treatment, cells were stained with MDC at a final concentration of 10 mM for 10 min at 37°C, collected and fixed in 3% paraformaldehyde in phosphate-buffered saline for 30 min. The cells were then trypsinised and analysed by flow cytometry using a FACScan cytometer and CellQuest software. For each condition, the percentage of cells with characteristic punctate MDC staining indicative of autophagy was assessed.

### Measurement of ROS Production

Intracellular ROS levels were measured using a DCFH-DA assay. Cells were incubated with DCFH-DA (5 mM) for 30 min at 37°C in 5% CO_2_ and then washed with HBSS. Total intracellular ROS levels were determined by FACS analysis of the oxidative conversion using a FACScan cytometer and CellQuest software.

### siRNA Assay

siRNAs targeting *ATG5* (human, sc-72578) *BECN1* (human, sc-29797), *P8* (human, sc-40792) and *CHOP/GADD153* (human, sc-35437) were purchased from Santa Cruz Biotechnology, along with a control siRNA (sc-44230). All siRNA transfections were performed with the Dharmafect 1 transfection reagent (Thermo Scientific, T-2001-03). Caco-2 cells were transfected with 100 nM siRNA for 24 hrs, and protein knockdown was then assessed by western blot analysis.

### Transmission Electron Microscopy

Caco-2 cells were collected and fixed in a solution containing 2% paraformaldehyde and 0.1% glutaraldehyde in 0.1 M sodium cacodylate for 2 hrs, postfixed with 1% OsO_4_ for 1.5 hrs, washed and stained in 3% aqueous uranyl acetate for 1 h. The samples were then washed again, dehydrated with a graded alcohol series, and embedded in Epon-Araldite resin (Canemco, #034). Ultrathin sections were cut on a Reichert ultramicrotome, counterstained with 0.3% lead citrate and examined on a Philips EM420 electron microscope.

### Confocal Microscopy

Caco-2 cells were transfected with the GFP-MAP1LC3B-expressing plasmid. After 24 hrs, cells were treated with oxaliplatin for 24 hrs. After infection, cells were washed with PBS and fixed by incubation in 4% paraformaldehyde for 10 min at 37°C. We used a Radiance 2000 laser scanning confocal microscope for confocal microscopy, followed by image analysis with LaserSharp 2000 software (Bio-Rad). Images were acquired in sequential scanning mode.

### Western Blot

Cells were washed with ice-cold PBS and then lysed in Triton X-100/glycerol buffer (50 mM Tris-HCl, 4 mM EDTA, 2 mM EGTA, 1 mM dithiothreitol and 25% wt/vol sucrose, pH 8.0, supplemented with 1% Triton X-100 and protease inhibitors). After centrifugation at 5,000×g for 15 min at 4°C, the protein concentration of the supernatant was measured with a BCA protein assay kit (Pierce, #23227). Lysates were separated using SDS-PAGE and transferred to polyvinylidene difluoride membranes. The membranes were blocked with 5% nonfat dry milk in Tris-buffered saline, pH 7.4, containing 0.05% Tween 20 (Sigma, P1379) and subsequently incubated with primary anti-human antibodies and horseradish peroxidase-conjugated anti-mouse antibodies (Jackson ImmunoResearch Laboratories, 115-035-003) or anti-rabbit secondary antibodies (Jackson ImmunoResearch Laboratories, 111-035-003) according to the manufacturer’s instructions. The proteins of interest were visualised using SuperSignal® West Dura substrate reagent (Thermo, #34080).

**Figure 5:**
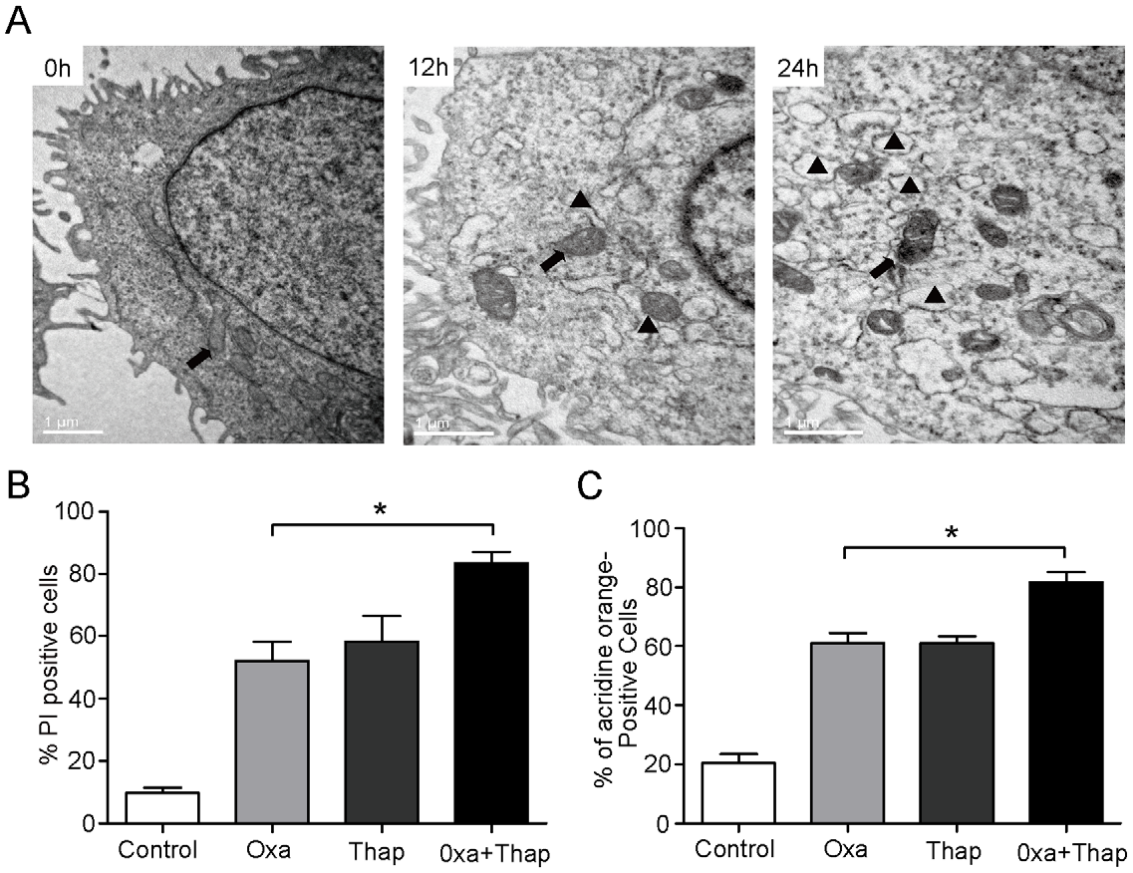
Oxaliplatin-induced cell death may be involved in ER stress. (A) Cells were treated with 10 µM oxaliplatin for 0, 12 and 24 hrs. Cells were directly fixed with 1% glutaraldehyde and postfixed with 2% osmium tetraoxide. Closed arrows indicate mitochondria, and closed triangles indicate the dilated ER. (B and C) Caco-2 cells were exposed to 200 nM thapsigargin (Thap), a combination of 200 nM thapsigargin, and 10 µM oxaliplatin (Oxa) or oxaliplatin alone for 24 h. Cell viability were analyzed with MTT. The cells that were positive for autophagosomes were detected using an acridine orange staining assay. *, *P*<0.05 for a chance difference between Oxa and Oxa +Thap.

**Figure 6:**
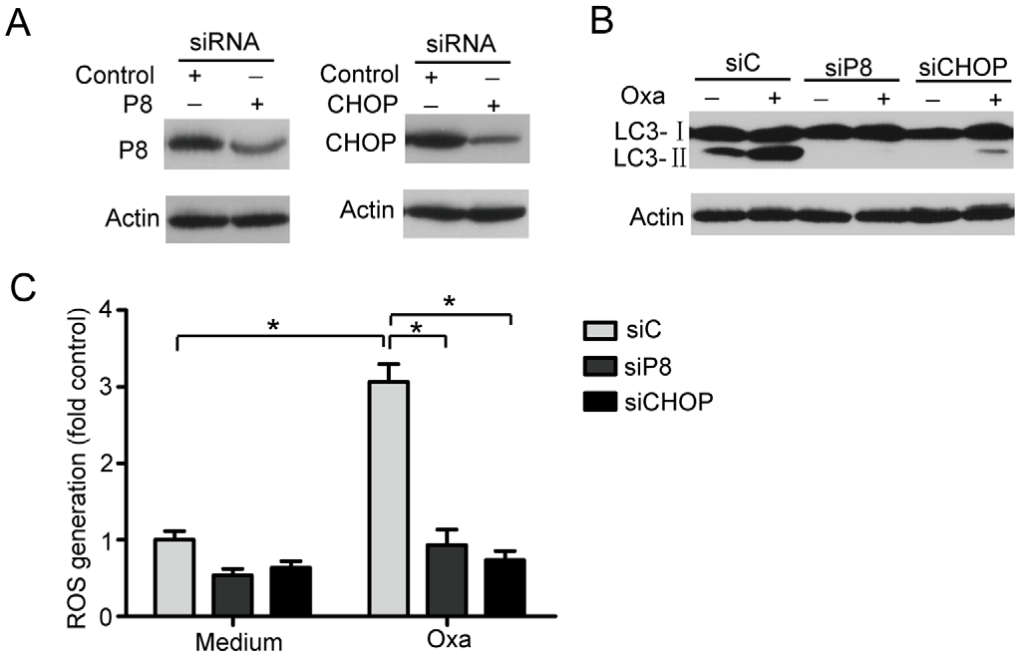
Oxaliplatin induces autophagy through ER stress. (A) The inhibition efficiency of siRNAs against P8 and CHOP. Caco-2 cells were transfected with siRNAs targeting P8 and CHOP (100 nM each) for 24 hrs, and the protein levels of the two targets were determined by western blot. (B) The effect of oxaliplatin (10 µM) on LC3B-II conversion in Caco-2 cells transfected with siC, siP8, or siCHOP. (C) Detection of ROS by flow cytometry in cells transfected with siC, siP8, or siCHOP (24 hrs). The data shown are the means and SD of at least 3 independent experiments. *, *P*<0.05.

### Cell Death Assay

Cells were trypsinised with 0.5 ml 0.25% trypsin for 3 min, collected and resuspended in 1 ml PBS. The cells were then incubated with 0.5 ml staining solution (10 µg/ml PI) at 37°C for 30 min in the dark. Cell death was detected by flow cytometry (BD FACScan Flow cytometer).

### Statistical Analysis

The results are expressed as the mean ± SD of at least 3 separate experiments performed in triplicate. The differences between groups were determined with the Mann-Whitney U-test using SPSS 13.0 software. Differences were declared significant at *P*<0.05. Statistically significant differences are indicated by asterisks (*P*<0.05 (*), *P*<0.01 (**)).

## Results

### Oxaliplatin Induces Cell Death in Human Colorectal Cancer Cells

To investigate the cell death induced by oxaliplatin in human colorectal cancer cells (Caco-2, SW1116 and HCT116), we first performed an MTT assay, which is considered an effective method for detecting cell death. As shown in Fig. 1A, there were dose-dependent decreases in relative viability after oxaliplatin treatment. Similar dose-dependent results were observed in cell death assays (Fig. 1B). These data indicate that oxaliplatin induces cell death in human colorectal cancer cells.

### Autophagy is Induced in Caco-2 Cells After Oxaliplatin Treatment

To determine whether autophagy could be induced after oxaliplatin treatment, we investigated the expression of MAP1LC3B (using Actin as a loading control), which is considered an accurate indicator of autophagy. We observed a gradual increase in the ratio of MAP1LC3B-II to Actin over time in cells treated with oxaliplatin compared to control cells (Fig. 2A). Furthermore, Baf A1 challenge resulted in the further accumulation of MAP1LC3B-II in Caco-2 cells after 24 hrs (Fig. 2A), suggesting that oxaliplatin promotes cellular autophagic flux. To further confirm that oxaliplatin induces autophagy in Caco-2 cells, we used a GFP- MAPLC3B puncta formation assay to monitor autophagy. As shown in Fig. 2B, oxaliplatin-treated Caco-2 cells displayed a significant increase in the percentage of cells with autophagosomes (GFP- MAPLC3B puncta) after 24 hrs compared with control cells (*P*<0.05) [16]. TEM of Caco-2 cells treated with oxaliplatin revealed a decrease in the number of autophagosomes (Fig. 2C). Similar results were obtained in acridine orange staining and MDC staining assays (Fig. 2D and 2E). The results above illustrate that oxaliplatin induces a complete autophagic response in Caco-2 cells.

### Autophagy Protects Against Oxaliplatin-induced Cell Death

Autophagy is enhanced under stress conditions, and it can promote cell survival or cell death depending on the type of cellular stress [17]. To determine whether oxaliplatin-induced autophagy promotes Caco-2 survival or death, we exposed the cells to autophagy inhibitors (3-methyladenine, 3-MA; bafilomycin A1, Baf A1) and activators (rapamycin, Rapa) and evaluated the subsequent cell death induced by oxaliplatin treatment. A significant increase in oxaliplatin-induced cell death was observed in Caco-2 cells after autophagy was inhibited (3-MA or Baf A1 treatment), while a decrease was observed in Caco-2 cells when autophagy was induced (Rapa treatment) (Fig. 3A and 3B). To further address the possibility that the inhibition of autophagy is responsible for the cell death induced by oxaliplatin, we assessed the effects of ATG5 and Beclin1 silencing by RNA interference (Fig. 3C). The siRNA-mediated knockdown of either ATG5 or Beclin1, which are required for autophagy, enhanced oxaliplatin-induced cell death (Fig. 3D and 3E), suggesting that autophagy protects against the cell death induced by oxaliplatin.

### Autophagy Induced by Oxaliplatin is Dependent on ROS, and Autophagy Decreases Oxaliplatin-induced ROS Generation

ROS generation is known to be associated with autophagy. We hypothesised that ROS production, which is a critical event in platinum-induced cell death, is the trigger for autophagy. Treatment with the ROS scavengers NAC and Tiron, which could decrease the effective level of ROS generation, suppressed the ratio of LC3B-II to Actin and the formation of acidic vesicular organelles (AVOs) (Fig. 4A and 4B), and increased cellular survival (Fig. 4C). This suggests that ROS promote the induction of autophagy. Moreover, to determine whether autophagy can modulate oxaliplatin-induced ROS generation, we quantified the intracellular levels of ROS in Caco-2 cells following a 24-hr treatment with 3-MA, Baf A1, Rapa, oxaliplatin, or a combination. As shown in Fig. 4D, there were marked increases in the generation of ROS in cells treated with oxaliplatin combination with 3-methylamphetamine or Baf A1 compared with cells treated with oxaliplatin alone. This result indicates that autophagy can decrease oxaliplatin-induced ROS generation.

### Oxaliplatin-induced Cell Death may be Involved in ER Stress

The above results indicated that ROS generation played an important role in oxaliplatin-induced cell death. Thus, it is necessary to elucidate the mechanism of ROS generation in oxaliplatin-induced cell death. The majority of ROS are generated by the leakage of electrons in the mitochondrial respiratory chain [18]. However, a recent investigation reported that ER dysfunction could be both a source and a consequence of increased ROS generation [13]. We analysed the ultrastructure of oxaliplatin-treated Caco-2 cells using electron microscopy. There were numerous dilated endoplasmic reticula present in oxaliplatin-treated Caco-2 cells compared to untreated cells (Fig. 5A). Moreover, Caco-2 cells were challenged with thapsigargin (an ER stress inducer). Thapsigargin challenge promoted cellular autophagic processes and cell death in Caco-2 cells (Fig. 5B and 5C). These findings indicate that oxaliplatin-induced cell death may be related to ER stress.

### Oxaliplatin Induces Autophagy through ER Stress

To confirm that ER stress is involved in regulating oxaliplatin-induced autophagy, we assessed the effect of P8 or CHOP/GADD153 silencing by RNA interference. As shown in Fig. 6A, both P8 and CHOP silencing drastically decreased the ratio of LC3B-II to Actin in oxaliplatin-treated Caco-2 cells (Fig. 6B). As expected, the levels of oxaliplatin-induced ROS generation were significantly decreased by P8 and CHOP siRNA (Fig. 6C). These results indicate that ER stress is upstream of autophagy and ROS generation in oxaliplatin-treated Caco-2 cells.

## Discussion

The results presented here suggest that oxaliplatin activates autophagy as a cytoprotective response via ER stress and ROS in human colorectal cancer cells. This novel model is supported by the following observations: i) oxaliplatin induces autophagy and cell death in colorectal cancer Caco-2 cells, ii) autophagy protects against oxaliplatin-induced cell death, and iii) autophagy induced by oxaliplatin is dependent on ROS and ER stress.

It is becoming increasingly recognised that altered autophagy is involved in the promotion or inhibition of cancer cell survival [19]. However, the molecular bases of the dual role of autophagy in cancer remain unknown. Experiments performed in oxaliplatin-treated Caco-2 cells showed that oxaliplatin, which induces cell death and enhances autophagy, concomitant with ROS generation, up-regulated ER stress. To directly evaluate the role of autophagy in this process, we assessed whether oxaliplatin-induced cell death was enhanced by the chemical and genetic inhibition of autophagy using 3-MA and Baf A1, which inhibit the first and last steps of the autophagy process, respectively, or RNA interference targeting the essential autophagy genes ATG5 and Beclin1. Remarkably, both 3-MA and Baf A1 treatment and ATG5 and Beclin1 siRNAs knockdown potentiated oxaliplatin-induced cell death. These findings indicate that autophagy directly contributes to the survival of Caco-2 cells treated with oxaliplatin.

It is of note that autophagy and ER stress are identified as novel mechanisms of drug resistance by several other studies [20], [21], [22]. Many stimuli, including common therapeutic agents, can promote autophagy in cancer cells. However, for most of these stimuli, disruption of autophagy does not enhance cell death. In our study, oxaliplatin activates autophagy as a cytoprotective response via ER stress and ROS in human colorectal cancer cells. An increasing number of studies indicate that autophagy is induced by ER stress in organisms from yeast to mammals [23]. Classic ER stress inducer, such as thapsigargin, promoted oxaliplatin-induced autophagy and cell death in Caco-2 cells (Fig. 5B and 5C). Consistent with this result, both P8 and CHOP silencing drastically decreased the ratio of LC3B-II to Actin in oxaliplatin-treated Caco-2 cells (Fig. 6B). These results support the idea that oxaliplatin-induced ER stress signals are critical for the induction of autophagy. These findings collectively provide a basis for future clinical trials to explore this combination therapy for the treatment of tumors resistant to targeted therapy.

ROS are highly reactive oxygen free radicals or non-radical molecules that are generated by multiple mechanisms; NOX and mitochondria are the major sources of ROS in cells [24]. These ROS serve as important multifaceted signalling molecules and regulate a number of cellular pathways, thus playing critical roles in determining cell fate [25]. Here, we showed that cell death and autophagy were promoted by the increase in intracellular ROS induced by oxaliplatin. This conclusion is supported by the observation that the radical scavengers NAC and Tiron inhibited oxaliplatin-induced autophagy. Autophagy is emerging as an important mediator of pathological responses, and the autophagy pathway engages in cross-talk with ROS and RNS (reactive nitrogen species) during both cell signalling and the protein damage response [26]. The inhibition of autophagy increases ROS generation in Caco-2 cells treated with the 3-MA/oxaliplatin or Baf A1/oxaliplatin combinations compared with cells treated with oxaliplatin alone. This result indicates that autophagy can decrease oxaliplatin-induced ROS generation. These findings strongly suggest that increasing ROS production and/or inhibiting autophagy may be a good therapeutic strategy for the management of colorectal cancer.

ROS generation is inseparably linked to mitochondrial dysfunction because mitochondria are both major generators and targets of reactive species [26]. The release of mitochondria-derived ROS into the cytosol induces apoptosis and also appears to be involved in the oxaliplatin-induced cell death pathway [22], [27]. D’Arango et al reported that colon cancer cells exposed to oxaliplatin were arrested in G2/M and underwent apoptosis [28]. Immunofluorescence staining demonstrated that the apoptotic cascade initiated by oxaliplatin is characterised by the translocation of Bax to the mitochondria and the release of cytochrome c into the cytosol [28]. These findings are consistent with a recent demonstration that oxaliplatin-induced apoptotic pathways include the loss of mitochondrial membrane potential and cytochrome c release, with the consequent activation of caspase-9 and caspase-3 [29]. Furthermore, our studies revealed that oxaliplatin-treated Caco-2 cells formed dilated endoplasmic reticula in a dose-dependent manner. The observation that P8 and CHOP silencing each drastically limited autophagy and decreased the levels of ROS generation in oxaliplatin-treated Caco-2 cells strongly supports the hypothesis that ER stress is a trigger of ROS generation, autophagy and apoptosis in these cells. Consistent with this hypothesis, oxaliplatin induced ER stress and mitochondrial perturbations in U2OS cells [30].

In conclusion, the results presented here provide evidence that oxaliplatin activates autophagy as a cytoprotective response via ER stress and ROS in human colorectal cancer cells. Although the detailed mechanisms through which the ER stress induced by oxaliplatin mediates ROS generation and autophagy remain to be elucidated, these findings provide important insight into the response of cancer cells to oxaliplatin.
